# Telmisartan Modulates the Oral Mucositis Induced by 5-Fluorouracil in Hamsters

**DOI:** 10.3389/fphys.2018.01204

**Published:** 2018-08-29

**Authors:** Maisie M. Barbosa, Aurigena A. de Araújo, Raimundo F. de Araújo Júnior, Gerlane C. B. Guerra, Gerly A. de Castro Brito, Renata C. Leitão, Susana B. Ribeiro, Emanuella de Aragão Tavares, Roseane C. Vasconcelos, Vinícius B. Garcia, Caroline A. C. X. de Medeiros

**Affiliations:** ^1^Post Graduation Program in Biological Sciences, Federal University of Rio Grande do Norte, Natal, Brazil; ^2^Post Graduation Program Public Health/Post Graduation Program in Pharmaceutical Science, Department of Biophysics and Pharmacology, Federal University of Rio Grande do Norte, Natal, Brazil; ^3^Post Graduation Program in Functional and Structural Biology/Post Graduation Program Health Science, Department of Morphology, Federal University of Rio Grande do Norte, Natal, Brazil; ^4^Post Graduation Program in Biological Sciences/Post Graduation Program in Pharmaceutical Science, Department of Biophysics and Pharmacology, Federal University of Rio Grande do Norte, Natal, Brazil; ^5^Post Graduation Program of Morphological Science, Department of Morphology, Universidade Federal do Ceará, Fortaleza, Brazil; ^6^Post Graduation Program in Biotechnology RENORBIO, Federal University of Rio Grande do Norte, Natal, Brazil; ^7^Post Graduation Program Public Health, Department of Dentistry, Federal University of Rio Grande do Norte, Natal, Brazil; ^8^Post Graduation in Program of Health Sciences, Federal University of Rio Grande do Norte, Natal, Brazil; ^9^Post Graduation Program in Biological Sciences/Post Graduation Program in Biotechnology RENORBIO, Department of Biophysics and Pharmacology, Federal University of Rio Grande do Norte, Natal, Brazil

**Keywords:** oral mucositis, telmisartan, 5-fluorouracil, hamsters, cytokines

## Abstract

Oral mucositis (OM) is a common adverse effect resulting from cancer therapy. The OM it has implications that may compromise oncologic treatment and decrease the patient’s quality of life. The therapeutic options to prevent or treat the symptoms of OM are scarce; there is no effective therapy that improves the symptoms. Based on the need for further research for the treatment of OM, the present study objective was to evaluate the effect of telmisartan (TELM) on the OM induced by 5-fluorouracil (5-FU), using as animal model Golden Syrian hamsters. 5-FU followed by mechanical trauma on day 4 was used to induce OM in hamsters. Euthanasia occurred on the day 10. The experiments were constituted by the groups saline, mechanical trauma, 5-FU, and TELM in three doses (1, 5, or 10 mg/kg). Macroscopic, histopathological, and immunohistochemical analyses as well as immunofluorescence experiments were performed on the oral mucosa of the animals. The samples also were used for analysis enzyme-linked immunosorbent assays and quantitative real-time polymerase chain reactions (qPCR). TELM (5 or 10 mg/kg) was able to reduce the inflammatory ulceration and infiltration in the oral mucosa of the animals, decreasing the levels of the cytokines TNF-α and IL-1β. These treatments was minimize the immunostaining for cyclooxygenase-2, matrix metalloproteinase-9, transforming growth factor-β, and smad 2/3. The nuclear transcription factor kappa B (NFκB) p65 and inducible nitric oxide synthase were reduced in the oral mucosa. Finally, TELM (10 mg/kg) increased the PPARγ gene expression and reduced STAT1 and NFκB p65 gene expression relative to the 5-FU group. Therefore, TELM prevents the OM produced by 5-FU on animal model.

## Introduction

Oral mucositis (OM) is a common effect associated with cancer treatments such as radiotherapy and/or chemotherapy (Sonis and Fey, 2002; Medeiros et al., 2011). This problem affects 20 to 40% of patients at the treatment of usual chemotherapy (Lima-Junior et al., 2014; Cidon, 2017) and occurs in practically all patients (90 to 97%) who receive radiotherapy in the head and neck region (Trotti et al., 2003). The inflammation in OM is characterized by the presence of ulceration and pseudomembranous formations in the oral cavity, oropharynx, or hypopharynx (Elting et al., 2008; Niscola, 2010). This complication compromises the patient’s quality of life because OM is extremely painful, interferes with food consumption, makes the patient more susceptible to infections and in some cases, may interfere with anticancer therapy (Lalla et al., 2008).

Chemotherapy and/or radiotherapy cause damage to the epithelial and submucosal cells, which leads resulting in apoptosis, atrophy and consequent ulceration (Sonis, 2007; Niscola, 2010; Maria et al., 2017). In the initial phase of OM, production of reactive oxygen species (ROS) is observed, which triggers various cellular signals that contribute to the lesion (Sonis, 2004). The nuclear transcription factor kappa B (NFκB) is the most well-characterized signaling pathway in the pathophysiology of OM. The NFκB increases the levels of proinflammatory cytokines as TNF-α and IL-1β, cyclooxygenase-2 (COX-2), matrix metalloproteinase-9 (MMP-9), inducible nitric oxide synthase (iNOS), and the transforming growth factor β (TGF-β)/SMAD 2/3 pathway (Sonis, 2004, 2009; Ribeiro et al., 2017). Proinflammatory cytokines amplify the tissue damage and the apoptosis of specially submucosal and basal epithelial cells (Ramirez-Amador et al., 2010). Macrophages are activated, which promote tissue damage by expressing metalloproteinases and producing additional TNF-α. TGF-β acts on serine threonine kinases that are coupled to the smad2/3 proteins. The TGF-β/smad2/3 pathway activates NFκB, promotes inflammation in the oral cavity and contributes to apoptosis (Sonis, 2007; Bian et al., 2015).

Oral mucositis is difficult to treat. Many substances have been used to treat or prevent OM, but there is not a definitive protocol (McGuire et al., 2013). Studies of the safety using cytokines and growth factor agents on the management in OM have been inconclusive (Raber-Durlacher et al., 2013). Thus, there is a need to identify agents that can improve OM. This article was objective to study the effect of telmisartan (TELM) on 5-fluorouracil (5-FU)-induced OM in Golden Syrian hamsters.

Telmisartan is an antihypertensive type 1 (AT1) receptor blocker (ARB) for angiotensin II (Ang II). It has pleiotropic anti-inflammatory effects, demonstrates benefits in atherosclerotic lesions in patients and is renoprotective (Hamano et al., 2011; Verdecchia et al., 2011). The renin-angiotensin-aldosterone system (RAAS) is knowing to acts in the regulate blood pressure, but authors have also demonstrated that Ang II regulates the expression of NFκB-dependent inflammatory genes (Jiang et al., 2004; Underwood and Adler, 2013). In OM, NFκB increases the expression of proinflammatory mediators (Sonis, 2004). TELM is well-tolerated and has a long half-life that allows blood pressure to be reduced for 24 h. Among the ARBs, TELM was the first drug approved to prevent cardiovascular events (Deppe et al., 2010).

## Materials and Methods

### Experimental Induction of Oral Mucositis (OM)

The 5-FU-induced MO model was performed as described by Sonis et al. (1990) and adapted by Leitao et al. (2007). Male Golden Syrian hamsters, 150–200 g, were experiment utilized. The experimental protocols were accepted in according to the Committee on Ethics in Animal Use (CEUA) of the Federal University of Rio Grande do Norte, under number 014/2016. OM was induced in the animals following the methodology of Sonis et al. (1990), by intraperitoneal (i.p.) administration of 5-FU, on the two first days of the experiment at doses of 60 mg/kg on 1st day and 40 mg/kg on 2nd day, followed by mechanical trauma (MT) on the 4th day of the experiment under anesthesia with 2% xylazine (10 mg/kg) and 10% ketamine (80 mg/kg) (i.p.), as adapted from de Araujo et al. (2015).

In according of the laboratory protocol, the MT was performed by exposing the right oral mucosa of the animal and the perforating the grooves with the tip of an 18 mm gauge needle across the oral mucosa, with the aim of enhancing OM and reproducing the clinical signs of chronic irritation that occur in patients during chewing. On the 10th day of the experimental model, after anesthesia with thiopental (100 mg/kg, i.p.) combined with 2% lidocaine i.p., they hamsters were euthanized, and specimens of the oral mucosa were retired for further analysis (Medeiros et al., 2011; de Araujo et al., 2015).

### Experimental Groups

The animals (*n* = 5 per group) were treated as follows: saline, without OM and treated with oral saline (p.o.); MT: the animals were subjected to MT but not 5-FU and treated with saline (p.o.); 5-FU: the animals received 5-FU and MT and were treated with saline (p.o.); TELM 1, 5, or 10: the animals received 5-FU and MT and were treated with TELM at doses 1, 5, or 10 mg/kg, orally (Araujo et al., 2013; Guerra et al., 2015). The TELM used was the commercial formulation (Micardis^®^). The tablet was dissolved in saline and administered 30 min prior to 5-FU, during the 10 days of the experimental model. The control groups received saline (p.o.) for 10 days.

### Macroscopic Analysis

Macroscopic analysis of the oral mucosa of the animals was performed as described by Sonis et al. (2000). Briefly, the inflammatory characteristics observed in the experimental model, such as erythema, erosion, vasodilatation, epithelial ulceration and abscess, were evaluated and classified according as value of the assigned score: 0 – completely healthy oral mucosa without erosion or vasodilation; 1 – presence of erythema, but no evidence of erosion of the oral mucosa; 2 – severe erythema, vasodilatation, and superficial erosion; 3 – ulcer formation on one or more mucosal surfaces but affecting no more than 25% of the cheek surface area, as well as severe erythema and vasodilatation; 4 – ulcers with a cumulative involvement of approximately 50% of the surface area of the cheek pouch and; 5 – virtually complete ulceration of the oral mucosa (Sonis et al., 2000; Lopes et al., 2010; Ribeiro et al., 2017).

### Histopathological Analysis

The samples of oral mucosa were initially fixed in 10% buffered formalin and then dehydrated and processed, followed by paraffin embedding. For study histopathological, were obtained sections of 5 μm thickness for hematoxylin-eosin staining. There sections were analyzed by light microscopy. The criteria analyzed were included infiltration of inflammatory cells, vasodilatation, presence of hemorrhagic areas, edema, ulcerations, and abscesses were independently scored following the following classification: 1 – normal epithelium and connective tissue without vasodilation, absence or discrete cellular infiltration or absence of hemorrhagic areas, ulcerations or abscesses; 2 – discrete areas of vasodilation or re-epithelialization, mild inflammatory infiltration with mononuclear prevalence and absence of hemorrhagic areas, edema, ulcerations, or abscesses; 3 – moderate vasodilatation, areas of epithelial degeneration, inflammatory infiltration with neutrophil prevalence, presence of hemorrhagic areas, edema and eventual ulceration, and absence of abscesses; 4 – severe vasodilation and inflammatory infiltration with neutrophils (Leitao et al., 2007, 2008; Watanabe et al., 2009; Medeiros et al., 2011; Skeff et al., 2014; de Araujo et al., 2015; Ribeiro et al., 2017).

### IL-1β and TNF-α Cytokine Assays

Samples of the oral mucosa were homogenized and processed based on the methodology of Kendall et al. (1983). In resume, the microplates were incubated overnight for 16 h by 4°C with antibodies against IL-1β and TNF-α, in according to the manufacturer’s protocol. Afterward, the plates were washed, and the wells were blocked, followed by addition of the samples and the standards in several dilutions, which were incubated for 2 h. The plates were washed three times with solutions containing bovine serum albumin (BSA), and then biotinylated anti-IL-1β or anti-TNF-α sheep polyclonal antibodies (diluted 1:1,000 in 1% BSA assay buffer) were added. Immediately after incubation at room temperature for 1 h, the plates were then washed again, and 50 mL of HRP-streptavidin (diluted 1:5,000) were added to each well. The *O*-phenylenediamine color reagent (50 mL) was added 15 min later, and the plates were incubated in the dark at 37°C for 15–20 min (Lima-Junior et al., 2012). The enzymatic reaction was quenched using H_2_SO_4_, and the absorbance was measured by means of UV-VIS spectrophotometry. These absorbance were determined at 490 nm and the results were presented as pg/mL (Lima-Junior et al., 2012; Araujo et al., 2013).

### Immunohistochemical Analysis for COX-2, MMP-9, TGF-β, and Smad2/3

For immunohistochemical study, samples of the oral mucosa were added to silanized slides to analysis (de Araujo et al., 2015). Separately, the section of tissue went through the processes of dewaxed, rehydrate, and washed with phosphate buffer saline (PBS) followed by the suppression of the endogenous peroxidase activity, using 3% hydrogen peroxide for blockade. In accordance with laboratorial protocol, the tissues samples were incubated, overnight, in primary antibodies (Santa Cruz Biotechnology, INTERPRISE, Brazil), at 4°C. Were used primary antibodies against COX-2 (1:400), MMP-9 (1:400) or TGF-β (1:400), and smad 2/3 (1:400) (Araujo Junior et al., 2016). For each one sections, were realized the wash with PBS and in followed, the incubation in streptavidin-HRP-conjugated secondary antibody (Biocare Medical, Concord, CA, United States), during 30 min (Araujo et al., 2013). The scores were assigned according to the intensity of the labeling and the amount of labeled cells, as follows: 1 – absence of labeling and/or labeled cells, 2 – poor labeling; 3 – moderate labeling; 4 – intense labeling (Ribeiro et al., 2017).

### Reverse Transcription Polymerase Chain Reaction (RT-PCR)

The extraction of total RNA from the oral mucosa samples, were realized using the TRIzol^TM^ reagent (Invitrogen, Co., United States) and the total RNA Isolation System S.V. (Promega, Madison, WI, United States). After extraction of the total RNA, was synthesized the first strand cDNA, from 20 μL of total RNA, using the ImProm-IITM reverse transcriptase system for RT-PCR (Promega), following the instructions to manufacturer’s (Chomczynski and Sacchi, 2006). The real-time quantitative PCR analyses of signal transducers and activators of transcription (STAT1), NFκB p65, and PPAR-γ mRNAs were realized using SYBR Green Mix with the Applied Biosystems^®^ 7500 FAST system (Applied Biosystems, Foster City, CA, United States). The primers were designed using Software Primer Express^TM^ version 3.0.1 (Applied Biosystems^TM^) and were as follows: STAT 1 (*Rattus norvegicus*) (Forward: 5′-ATT AAC GAT GAG TTA GTG GAG TGG AA-3′; Reverse: 5′-GGT GGC CCC CCG ATA C-3′); NFκB p65 (*Mesocricetus auratus*) (Forward: 5′-GAA GAA GCG AGA CCT GGA GCA A-3′; Reverse: 5′-GTT GAT GGT GCT GAG GGA TGC T-3′); PPARγ (*M. auratus*) (Forward: 5′-CAT GAC CAG GGA GTT CCT CAA-3′; Reverse: 5′-TGG GCT CCA TAA AGT CAC CAA-3′); GAPDH (*M. auratus*) (Forward: 5′-AAC TTT GGC ATC GTG GAA GG-3′; Reverse: 5′-GTG GAT GCA GGG ATG ATG TTC-3′). The plates were prepared with 10 μL final volume in each well (2.0 μL of cDNA + 8.0 μL of the prepared mixture containing the SYBR Mix, nuclease-free water and forward and reverse primers) and were added in the StepOnePlus thermal cycler (Applied Biosystems^TM^, United States) according to the manufacturer’s protocol. For calculate relative levels obtained in this experiment, were used the mean Ct values. This value was contributing to calculate target genes for each of the submitted groups induced OM for 5-FU, when compared to the negative control group (group without OM). Expression data were standardized using to the GAPDH reference gene in the formula 2^-ΔΔC_t_^ (Rao et al., 2013).

### Immunofluorescence for iNOS and NFkB p65

Immunofluorescence was realized according to the previously described methodology of Guerra et al. (2016) and Ribeiro et al. (2017). Sections of the oral mucosa from the hamsters, three per group, were dewaxed using xylol and, posteriorly, washed in concentrations different of ethanol and PBS. In process of antigen retrieval, was utilized a 10 mM sodium citrate solution containing 0.05% Tween 20 for a period 40 min at 95°C. Posteriorly, the samples were incubated in Sudan-Black 0.1% diluted in 70% ethanol, for 40 min, at room temperature to decrease the tissue autofluorescence. The slides again were incubated at 4°C overnight with mouse monoclonal anti-NFκB (1:200; sc-8008, Santa Cruz Biotechnology) or rat polyclonal anti-iNOS (1:200, sc-8332, Santa Cruz Biotechnology) primary antibodies and were washed three times for 5 min in PBS containing 0.2% Triton X-100. Subsequently, the samples were incubated with an Alexa Fluor 488-conjugated goat anti-rabbit secondary antibody (1:500 in 1% BSA blocking solution; Dako, Brazil, and United States). Finally, coverslips were applied using VECTASHIELD^®^ mounting medium with DAPI (4′,6-diamidino-2′-phenylindole). Immunofluorescence images were obtained using a Carl Zeiss Laser Scanning microscope (LSM 710, 20× objective, Carl Zeiss, Jena, Germany) (Araujo Junior et al., 2016; de Assis et al., 2016).

### Statistical Analysis

The data are presented as the mean ± standard deviation or as the median ± standard deviation. The analysis of variance (ANOVA) and Tukey’s post-test were used to compare the means. To compare the medians, the Kruskal–Wallis test followed by Dunn’s test was used. The minimum significance was set at *p* < 0.05, and the analyses were performed using the Prism program, version 6.0 GraphPad Software, La Jolla, CA, United States.

## Results

### Macroscopic Aspects and Histopathological Analysis

**FIGURE 1:**
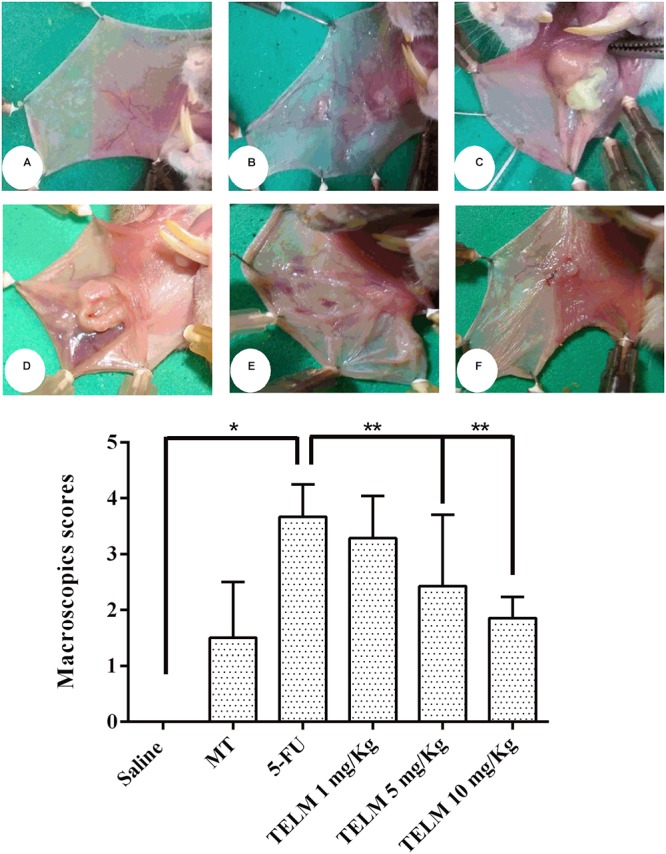
Macroscopic analysis and scores of the oral mucosa of hamsters with oral mucositis (OM) induced by 5-fluorouracil (5-FU) and MT. The saline group consisted of animals with healthy oral mucosa **(A)**. The mechanical trauma (MT) group demonstrated vasodilation and erythema in the jugal mucosa **(B)**. The 5-FU group had changes in the oral mucosa, erythema, vasodilatation, erosion, and extensive ulcers **(C)**. TELM at 1 mg/kg did not improve the OM **(D)**. The groups treated with TELM 5 **(E)**, or 10 mg/kg **(F)** had mild vasodilation and superficial erosion in the oral mucosa, with no evidence of ulcers. The scores are represented with the standard error of the mean (*n* = 5). ^∗^*p* < 0.05 vs. the saline group, ^∗∗^*p* < 0.05 vs. the 5-FU group (Kruskal–Wallis test and Dunn’s multiple comparison test).

**FIGURE 2:**
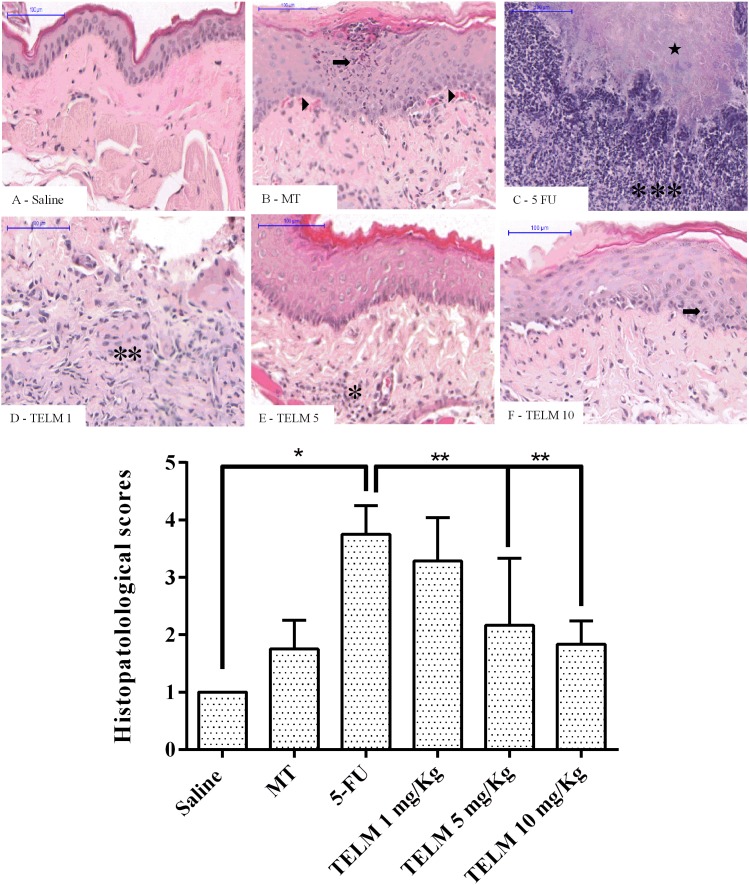
Histopathological analysis and scores of the oral mucosa of hamsters with oral mucositis (OM) induced by 5-fluorouracil (5-FU). The saline group consisted of animals with healthy oral mucosa **(A)**. The mechanical trauma (MT) group demonstrated areas of epithelial hyperplasia exhibiting foci of moderate exocytosis (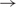), small-caliber blood vessels (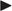), and inflammatory cells dispersed by connective tissue **(B)**. The 5-FU group had alterations in the oral mucosa including intense inflammatory infiltrates (^∗∗∗^), erosion and extensive ulcers **(C)**, and presence of purulent exudate, with areas suggestive of necrosis (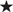). TELM at 1 mg/kg did not improve the OM and presented ulcerated areas and moderate inflammatory infiltrate (^∗∗^) **(D)**, however, TELM 5 **(E)** or 10 mg/kg **(F)** showed vasodilatation and discrete cell infiltration (^∗^), the absence of hemorrhage, edema, ulcers, and abscesses. The scores are presented as the medians ± standard error of the mean (*n* = 5). ^∗^*p* < 0.05 vs. the saline group, ^∗∗^*p* < 0.05 vs. the 5-FU group (Kruskal–Wallis test and Dunn’s multiple comparison test).

In analyze oral mucosa of the animals belonging saline group had no macroscopic or histopathological changes (**Figure 1A**; with score 0 and **Figure 2A**; score of 1). On MT group animals showed some changes. Macroscopic aspects demonstrated in these group, included presence of erythema and with absence of mucosal erosion (**Figure 1B**; value of score equal 1), already in the histopathological aspects were observed vasodilatation discrete and cell infiltration, areas of re-epithelialization, and absence of hemorrhage, edema, ulcers, and abscesses (**Figure 2B**; with score of 2). The aspects observed of the 5-FU group demonstrated ulcers in approximately 50% of the area of the oral mucosa the animals euthanized (**Figure 1C**; score of 4, *p* < 0.05), as others technical features including severe vasodilatation, and inflammatory infiltration with neutrophils, abscesses, and extensive ulcers (**Figure 2C**; score of 4, *p* < 0.05). These aspects were compared to the saline group. TELM at 1 mg/kg did not prevent the OM lesions, compared to the 5-FU group (**Figures 1D**, **2D**; score of 3). The groups that had been treated with TELM at 5 or 10 mg/kg has aspects as discrete vasodilatation, areas of re-epithelialization, discrete cell infiltration, and absence of hemorrhage, edema, ulcers, and abscesses (**Figures 1E,F**, **2E,F**; score of 2, *p* < 0.05). These latter groups the observed results were compared with the experimental 5-FU group.

### TNF-α and IL-1β Cytokines

**FIGURE 3:**
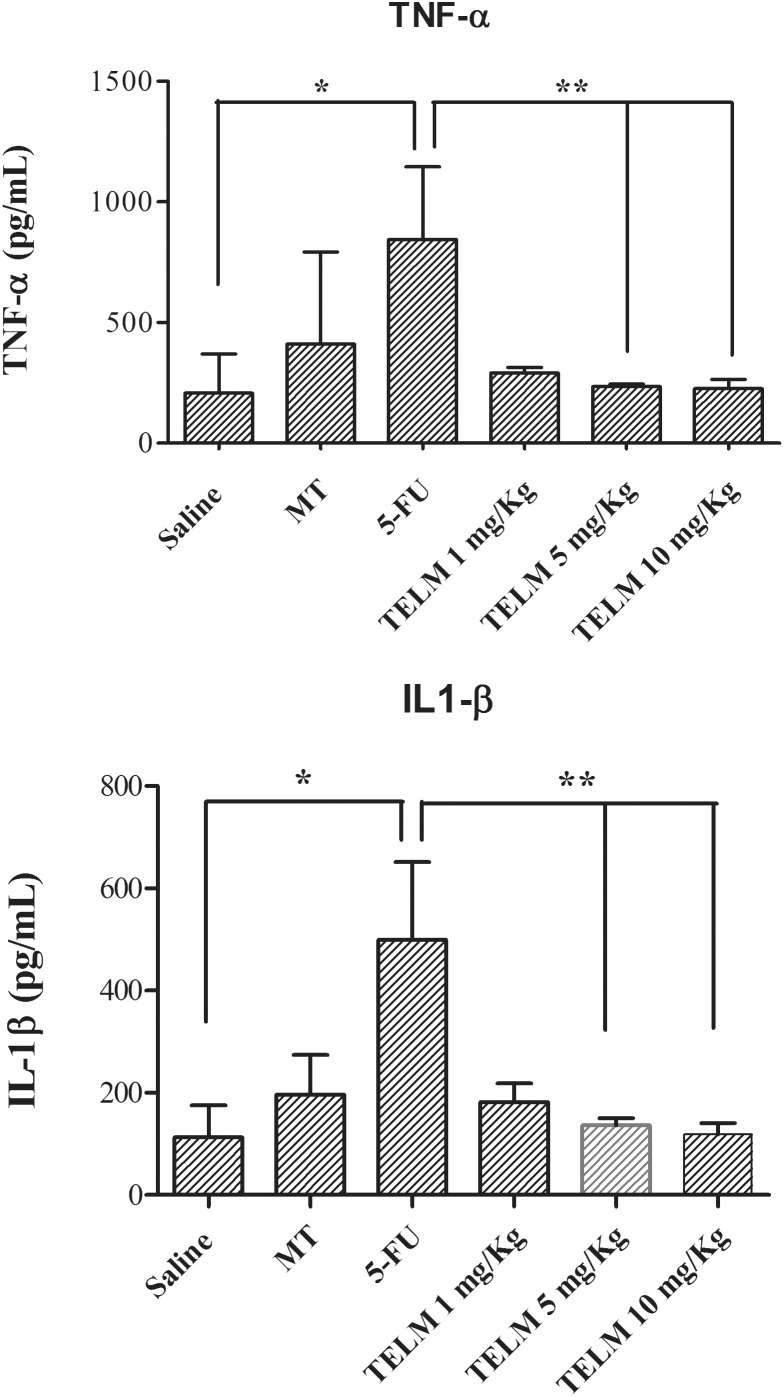
Levels of tumor necrosis factor (TNF)-α and interleukin (IL)-1β cytokines in the oral mucosa of hamsters with oral mucositis (OM). The saline group consists of animals without OM. The mechanical trauma (MT) group consisted of hamsters that received excoriations of the oral mucosa, without 5-fluorouracil (5-FU). The 5-FU group received 5-FU, was subjected to MT and was treated with saline i.p. The TELM groups received 5-FU, were subjected to MT, and received TELM i.p. at one of three doses (1, 5, or 10 mg/kg). The results are presented as the mean ± standard error of the mean (*n* = 5). ^∗^*p* < 0.05 vs. the saline group, ^∗∗^*p* < 0.05 vs. the 5-FU group (analysis of variance with Tukey’s post-test).

The analyze for cytokines demonstrated the 5-FU group had higher values of the TNF-α (**Figure 3**, *p* < 0.05) and IL-1β (**Figure 3**, *p* < 0.05), contrary result than with exhibited in the saline group. TELM at doses of 5 or 10 mg/kg reduced the levels of TNF-α (**Figure 3**, *p* < 0.05) and IL-1β (**Figure 3**, *p* < 0.05), when the results obtained in this group were compared to the levels found in the 5-FU group. The results get by TELM at a dose of 1 mg/kg did not reduce the levels of proinflammatory cytokines compared to the 5-FU group.

### Immunohistochemical Analyses (MMP-9, COX-2, TGF-β, and Smad 2/3)

**FIGURE 4:**
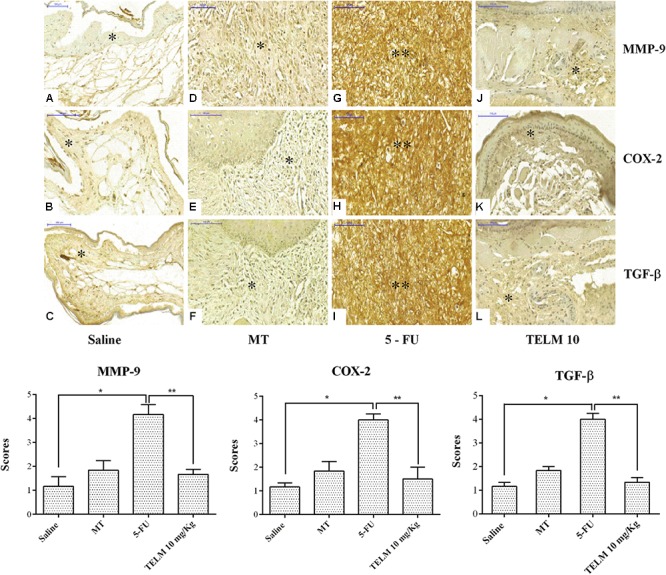
Immunohistochemistry and scores for matrix metalloproteinase-9 (MMP-9), cyclooxygenase-2 (COX-2), and transforming growth factor beta (TGF-β). The saline group **(A**–**C)** and the mechanical trauma (MT) group **(D**–**F)** had little immunostaining. The inflammatory cells and fibroblasts of the jugal mucosa of the 5-FU group were intensely labeled for MMP-9 **(G)**, COX-2 **(H)**, and TGF-β **(I)** compared to the saline group. TELM at 10 mg/kg reduced the immunostaining for MMP-9 **(J)**, COX-2 **(K)**, and TGF-β **(L)**, compared to the 5-FU group. The scores are represented as the medians ± standard error of the mean (*n* = 5). ^∗^*p* < 0.05 vs. the saline group, ^∗∗^*p* < 0.05 vs. the 5-FU group (Kruskal–Wallis test and Dunn’s multiple comparison test).

**FIGURE 5:**
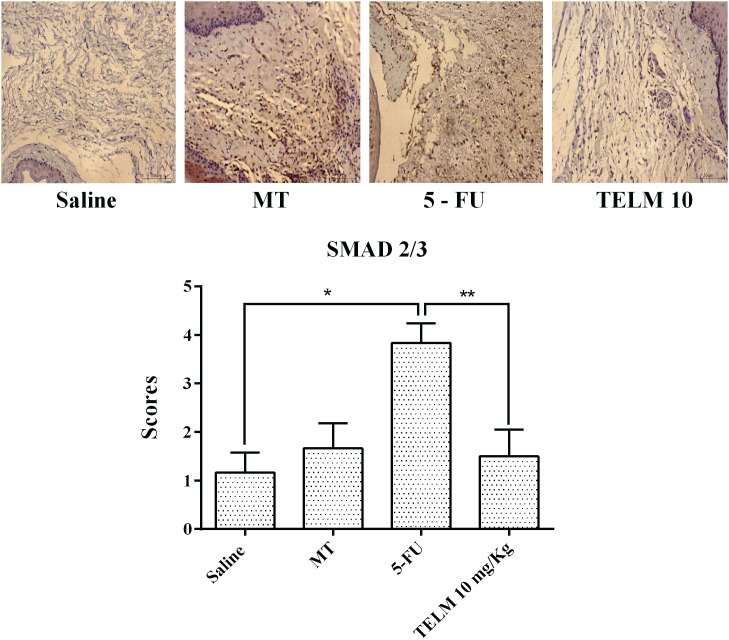
Immunohistochemistry and scores for Smad 2/3. The saline group and the mechanical trauma (MT) group had little immunostaining. The inflammatory cells and fibroblasts of the jugal mucosa of the 5-FU group were intensely labeled Smad 2/3 compared to the saline group. TELM at 10 mg/kg reduced the immunostaining for Smad 2/3, compared to the 5-FU group. The scores are represented as the medians ± standard error of the mean (*n* = 5). ^∗^*p* < 0.05 vs. the saline group, ^∗∗^*p* < 0.05 vs. the 5-FU group (Kruskal–Wallis test and Dunn’s multiple comparison test).

The analyzes realized with the oral mucosa of the animals with OM induced for 5-FU (*n* = 4), demonstrated intense marking for proteins MMP-9 (**Figure 4G**; score of 4, *p* < 0.05), COX-2 (**Figure 4H**; score of 4, *p* < 0.05), TGF-β (**Figure 4I**; score of 4, *p* < 0.05), and Smad 2/3 (**Figure 5**, score of 4, *p* < 0.05), compared with samples of the saline group (**Figures 4A–F**). Inversely, the TELM (10 mg/kg) group showed low immunostaining for MMP-9 (**Figure 4J**; score of 2, *p* < 0.05), COX-2 (**Figure 4K**; score of 2, *p* < 0.05), TGF-β (**Figure 4L**; score of 2, *p* < 0.05) and Smad 2/3 (**Figure 5**; score of 2, *p* < 0.05), also compared with the 5-FU group.

### qPCR for STAT 1, NFκB p65, and PPARγ

**FIGURE 6:**
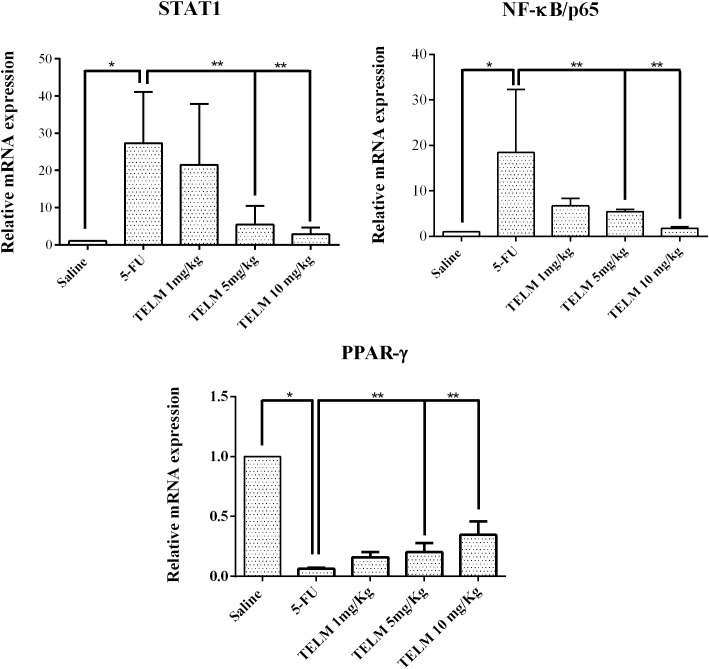
Real-time polymerase chain reaction for the signal transducer and transcription activator 1 (STAT1), nuclear factor NFκB p65 (NFκB p65) and peroxisome proliferator-activated receptor gamma (PPARγ). 5-Fluorouracil (5-FU) increased the expression of the STAT1 gene and nuclear factor NFκB p65, compared to the saline group. Telmisartan at 10 mg/kg increased the PPARγ gene expression and decreased the expression of STAT1 and NFκB p65 compared to the 5-FU group (*n* = 5). ^∗^*p* < 0.05 compared to saline group; ^∗∗^*p* < 0.05 compared to the 5-FU group; analysis of variance with Tukey’s post-test.

The quantitative real-time polymerase chain reactions (qPCR) experiment for exhibited 5-FU group had a reduced mRNA expression of PPARγ when compared to the saline group (**Figure 6**; *p* < 0.05), besides there was an increased mRNA expression of NFκB p65 or STAT 1 (**Figure 6**; *p* < 0.05). TELM at 10 mg/kg increased the mRNA expression of PPARγ and demonstrated reduce in the mRNA expression for NFκB p65 or STAT 1 comparing to the 5-FU group (**Figure 6**, *p* < 0.05).

### Immunofluorescence for iNOS and NFκB p65

**FIGURE 7:**
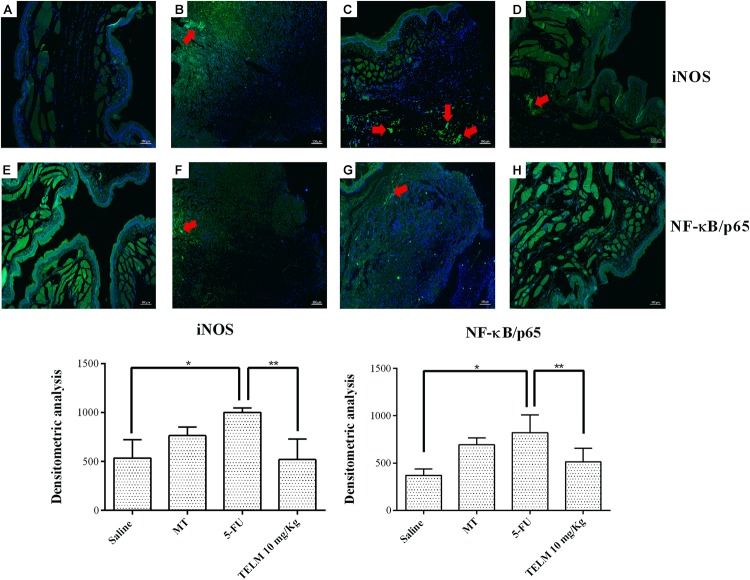
Immunofluorescence for iNOS; NFκB p65 and mean of the densitometric analysis. The 5-fluorouracil (5-FU) group **(C**,**G)** had higher green labeling than that of the mechanical trauma (MT) group **(B**,**F)** or the saline group **(A**,**E)** (*p* < 0.05; *n* = 5). The TELM 10 mg/kg group **(D**,**H)** had a reduced immunoreactivity compared to the 5-FU group (*n* = 5; ^∗^*p* < 0.05 vs. the saline group, ^∗∗^*p* < 0.05 vs. the 5-FU group (analysis of variance with Tukey’s post-test).

The research for the cellular iNOS and NFκB p65 labeling (green) was strong and diffuse in the 5-FU group (**Figure 7C**), moderate in the MT group (**Figure 7B**), and weak in the saline group. TELM (10 mg/kg) group also presented weak immunostaining (**Figures 7A,D**, respectively). The densitometric analysis confirmed that the iNOS and NFκB p65 immunoreactivity was significantly increased in the 5-FU group, relative to the saline group (**Figure 7**; *p* < 0.05). The TELM (10 mg/kg) group had a reduced immunoreactivity compared to the 5-FU group (**Figure 7**; *p* < 0.05).

## Discussion

In the present study, TELM at 5 or 10 mg/kg prevents the OM induced by 5-FU and TM, as indicated by the reduced levels of inflammatory infiltrates, hemorrhagic areas, vasodilation, and absence of abscesses and ulcers observed in the macroscopic and histopathological analyses of the oral mucosa. The anti-inflammatory effect of TELM has been demonstrated in the literature in other experimental models (Czechowska et al., 2016; Li et al., 2016).

The NFκB signaling pathway is the further most important and most utilized in studying pathway in the pathophysiology of MO. Chemotherapy with 5-FU promotes direct apoptosis in the basal epithelial cells and submucosal cells of the oral cavity tissue (Ribeiro et al., 2017). NFκB is activated, which results in the subsequent production of the proinflammatory cytokines TNF-α and IL-1β. TNF-α interacts synergistically with NFκB, amplifying its activation. NFκB also increases the expression of other proinflammatory factors, thus contributing to the tissue injury (Li and Lin, 2008).

In the present study, TELM reduced the protein and gene expression of NFκB p65 as well as values of the cytokines pro-inflammatory as the TNF-α and IL-1β. TELM inhibits the activity of Ang II on the AT1 receptor (AT1R). Ang II acts on the AT1R or AT2R. The role of the AT2R receptor is not completely defined, but it inhibits cell growth and the infiltration of inflammatory cells into the kidney. However, the action of Ang II on AT1R triggers the expression of several proinflammatory genes such as NFκB and activates the TGF-β/Smad2/3 signaling in renal injury (Ruster and Wolf, 2011).

This research, the 5-FU group had raise immunoblotting signals for NFkB p65 and TGF-β and increased expression of the Smad2/3 protein. TELM reduced the expression of the TGF-β/Smad2/3 signaling pathway. Consistent with the present results others have demonstrated that TELM attenuates the production of TGF-β in experimental hepatic fibrosis (Attia et al., 2013; Czechowska et al., 2016). TGF-β signaling participates in the pathophysiology of OM by inhibiting epithelial cell growth and causing apoptosis as well as by promoting the activation of the transcription factor NFκB (Massagué, 2008). Smad 7 improves the OM of patients undergoing chemotherapy or radiotherapy by inhibiting the TGF-β/Smad2/3 and NFkB signaling (Han et al., 2013). Thus, blockade of NFκB and TGF-β signaling may explain part of the beneficial effect of TELM on OM in hamsters.

The nuclear transcription factor kappa B induces transcription of MMP-9, COX-2, and iNOS (Sonis, 2007). TELM at 10 mg/kg reduced the immunostaining for COX2, MMP-9, and iNOS in the oral mucosa of hamsters compared to the 5-FU group. MMPs are enzymes that were initially studied in the context of their roles in regulating the homeostasis of the extracellular matrix components and maintaining the physiology of healthy oral mucosa (Clark et al., 2008). However, MMP-9 is involved in the chemotherapy-induced OM stages, including inflammatory cell migration, apoptosis, cytokine activation, and dysregulation of normal cell kinetics, which suggests that it participates in the ulcerative phase (Al-Azri et al., 2015). Like the results in present study, others search have demonstrated that TELM downregulates MMP-9 and COX2 (Araujo et al., 2013).

The participation of nitric oxide (NO) in the OM induced by 5-FU is consistent with previous reports showing that cytokines TNF-α and IL-1β stimulate NO production from iNOS (Liu et al., 2005). Other authors have demonstrated that the NO produced by iNOS is associated with the damage and inflammatory events of 5-FU-induced OM (Leitao et al., 2007). Consistent with the results of the current study, TELM decreased the iNOS activity, thus inhibiting the excessive NO generation and inflammatory responses in other models of inflammation (Yuan et al., 2015).

Telmisartan at 10 mg/kg increased the gene expression of PPARγ (peroxisome proliferator-activated receptor-gamma) compared to the 5-FU group. Similarly, others have demonstrated that TELM increases the gene expression of PPARγ in an *in vitro* human umbilical vein endothelial cell model and decreases the homocysteine-induced inflammatory response in endothelial cells (Xu et al., 2014). The PPARs are a family of nuclear receptors that regulate the immune and inflammatory response. Among the ARBs, TELM is the most potent agonist for PPARγ: it activates the receptor to approximately 25–30% of the maximum level reached by full agonists such as pioglitazone and rosiglitazone (Janke et al., 2006).

Some investigators have demonstrated that part of the anti-inflammatory effect of PPARγ is mediated by direct inhibition of the NFκB p65 (Chung et al., 2000) as well as by the inhibition of STAT (Ji et al., 2005). In the current study, TELM inhibited NFκB p65 and reduced the STAT 1 gene expression compared to the 5-FU group. Seven members of the STAT protein family have been described, and STAT1 activates iNOS (Ohmori and Hamilton, 2001). 5-FU increased the STAT 1 gene expression and inhibited the PPARγ gene expression in the present study. However, studies of the effects on the expression of these proteins are necessary to confirm the participation of STAT 1 and the PPARγ gene in the mechanisms associated with the protective effects of TELM against 5-FU-induced OM.

## Conclusion

**FIGURE 8:**
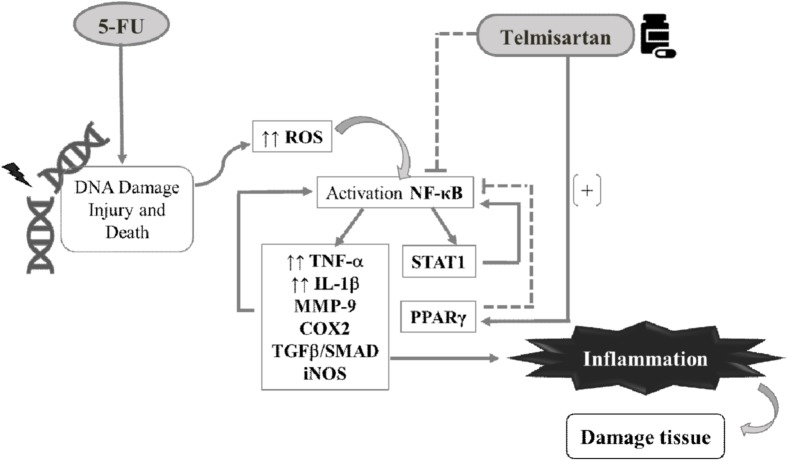
Pharmacological modulation of the oral mucositis induced by 5-fluorouracil (5-FU) by telmisartan. DNA damage due to 5-FU activates NFκB, and this nuclear factor induces the expression of proinflammatory cytokines such as IL-1β and TNF-α which promote inflammation in the oral mucosa and tissue damage. Telmisartan interferes with NFκB p65 to decrease IL-1β, TNF-α, MMP-9, COX2, TGF-β, Smad 2/3, and iNOS improving oral mucositis. Telmisartan reduces NFκB by decreasing STAT 1 gene expression, in addition to increasing the gene expression of PPARγ. ROS, reactive oxygen species; NFκB p65, nuclear transcription factor kappa B; STAT 1, signal transducer and activator of transcription 1; PPARγ, peroxisome proliferator-activated receptors.

TELM prevents the 5-FU-induced OM lesions by reducing the expression of the proinflammatory mediators TNF-α, IL-1β, TGF-β, Smad 2/3, COX2, MMP9, and iNOS, by inhibiting NFκB, by increasing the PPARγ gene expression, and by reducing the STAT1 gene expression. These results suggest the contribute these pathways in the protecting outcome that TELM present in Oral Mucosa (**Figure 8**). Though, more researches are needed to elucidate these findings.

## Author Contributions

CdM: investigation, editing of the manuscript, supervision, and validation. MB and AdA: investigation, preparation of the manuscript, analyzes, methodologies, and validation. RdAJ and GG: investigation and editing of the manuscript and supervision. GdCB and RL: editing and revision of the manuscript. SR, EdAT, RV, and VG: analyzes and methodologies.

## Conflict of Interest Statement

The authors declare that the research was conducted in the absence of any commercial or financial relationships that could be construed as a potential conflict of interest.
